# Bridging the occupational policy to practice gap: user-centered designed toolbox talks for landscaping tree care

**DOI:** 10.3389/fpubh.2025.1690149

**Published:** 2025-11-28

**Authors:** Gregory D. Kearney, Guiseppe Getto, Jamie Hisel

**Affiliations:** 1 Department of Public Health, Brody School of Medicine, East Carolina University, Greenville, NC, United States; 2 Department of Human-Centered Information Design and Technology, School of Engineering, Mercer University, Macon, GA, United States

**Keywords:** health promotion, injury prevention, occupational health, small business, community-based participatory research

## Abstract

**Introduction:**

Landscaping and tree care work are among the most dangerous jobs in the U.S. with fatality rates more than five times the national average, and injury rates twice as high. Despite these alarming statistics, these occupations remain largely unregulated, with little safety training information available for workers.

**Aim:**

This study aimed to address this gap by developing culturally relevant, policy-driven, safety “Toolbox Talks,” tailored to the landscaping and tree care industry. The specific objectives were to evaluate the usability of prototype Toolbox Talks, and validate their clarity, applicability, and usefulness for small business environments.

**Methods:**

This was a mixed-methods study that involved workers and supervisor participants (*N* = 60) from small landscaping and tree care companies. Prototype Toolbox Talks were developed and field tested with participants. User “pain points,” or issues related to design layout, terminology, graphics, were identified in group sessions, and revised based on iterative testing.

**Results:**

In final testing sessions, workers (*n* = 37) and supervisors (*n* = 23) consistently described Toolbox Talks being “highly needed” and “relevant” for addressing workplace hazards and promoting safe practices. Narrative “true story” examples strongly resonated with workers, reinforcing hazard recognition, and prevention strategies. Supervisors reported increased confidence in delivering Toolbox Talks, noting improved clarity during trainings. The use of Spanish terminology and phrasing enhanced accessibility for Spanish-speaking participants.

**Conclusion:**

Engaging users directly in the development process significantly improved the relevance, clarity, cultural fit, and alignment of Toolbox Talks with the needs of landscaping and tree care workers and supervisors. This participatory approach enhanced usability and demonstrated that Toolbox Talks can serve as a practical, scalable model for strengthening safety communication in high-risk occupations with limited regulatory oversight.

## Introduction

In 2024, the U.S. Bureau of Labor Statistics (BLS) reported that landscaping and groundskeeping workers experienced the highest number of fatalities in this sector, followed by tree trimmers and pruners. Although these workers make up less than 1% of the U.S. labor force, their fatal occupational injury rates consistently rank among the highest nationwide (1–3). In 2021, the fatality rate in the landscaping industry was 25.1 deaths per 100,000 full-time workers, more than six times the average of 3.8 deaths per 100,000 (1). These persistently high rates underscore the urgency to provide clear and culturally relevant safety communication to workers. Yet, despite these alarming statistics, many workers operating as small businesses in these high-hazardous occupations often lack the administrative capacity, resources, or technical expertise necessary to create compliant training materials tailored to their workforce.

Tailgate Safety Talks, or Toolbox Talks (TBTs) offer great, low-cost, and effective opportunities to raise safety awareness to workers prior to performing daily tasks to landscaping and tree care workers (4). In general, TBTs are informal safety discussions lasting 5 to 10 min, typically led by supervisors at the beginning of the workday or prior to undertaking high-risk tasks. Commonly utilized across various industries, TBTs can serve as a valuable mechanism for reinforcing occupational safety, training requirements, and enhancing communication between supervisors and workers (5).

In a recent scoping review of TBT studies (*n* = 14) and their effectiveness as an intervention, the authors identified positive findings, including increased, worker safety knowledge. Among key characteristics of TBTs that enhanced effectiveness identified were, tailoring to address specific job-related hazards (6); training for supervisors on how to deliver TBTs effectively (7); active engagement of workers as part of the training (6); inclusion of relatable, real-life injury narratives (8); and delivering TBT content in the worker's primary language (9, 10).

Research studies in the U.S. and internationally has consistently shown that small enterprises in the landscaping and tree care sectors experience disproportionately high rates of occupational injuries and fatalities, including incidents involving falling trees, electrocutions, overexertion, and the use of sharp tools and powered equipment (11). These elevated risks are often linked to inadequate safety management systems, minimal commitment to OSH by business owners, and a lack of OSH support services (12). In 2023-24, there were an estimated 1.3 million landscaping and tree care workers employed across an estimated 750,000 businesses in the U.S., of which the vast majority were small, family-owned or owner-operated enterprises (13, 14). This large, predominantly low wage workforce, exacerbates ongoing labor challenges, which are often marked by high employee turnover and a heavy reliance on seasonal or temporary workers; many of whom have little or no prior training or experience in the industry (3, 15).

To address persistent labor shortages, U.S. employers have increasingly relied on the H-2B guest worker program, a federal visa program that allows employers to hire foreign workers for temporary, non-agricultural jobs when domestic labor is unavailable. Between 2018 and 2023, employer demand for H-2B visas rose by 46%, with over 215,000 petitions submitted by approximately 8,000 employers (16, 17). In 2024, the landscaping industry accounted for the largest share of H-2B visa use, representing more than half of the 66,000 visas issued nationwide (18). While the H-2B program helps alleviate labor shortages, it also introduces unique OSH challenges. Most H-2B workers originate from Mexico and Latin American countries such as El Salvador, Guatemala, and Honduras, bringing with them diverse cultural backgrounds, limited English proficiency, and often minimal prior safety training (19). These factors can significantly reduce the effectiveness of conventional safety programs, especially when training materials are not translated or culturally adapted. When combined with the structural limitations typical of small businesses, such as limited administrative support and high workforce turnover, these communication barriers contribute to reduced hazard awareness, inconsistent safety messaging, and increased risk of injury. Adapting safety training tools, such as TBTs, to be both culturally responsive and linguistically accessible represents a practical, evidence-based strategy to enhance and improve OSH outcomes among this vulnerable labor force.

Supervisors play a crucial role in connecting training content with daily operations, making sure safety materials are practical, relevant, and easy to apply (20). When supervisors are actively involved in creating and delivering training, they can customize content to reflect real-world situations, which helps workers understand and use it better. Providing supervisors with clear, user-friendly tools also ensures consistent messaging and strengthens safety responsibility within teams. The supervisory role is especially vital in high-risk, labor-heavy industries, where effective engagement between workers and supervisors is a key factor in the success of occupational safety and health (OSH) training (21). Disengaged workers are more likely to misinterpret safety information, take shortcuts, and bypass safety procedures, all of which increase the risk of injury and non-compliance (20–22). Disengaged workers are also less likely to report hazards or take part in proactive safety initiatives (3, 22). Studies show that training designed without input from frontline workers or supervisors often fails to address jobsite realities, cultural differences, or language barriers (22, 23). On the other hand, collaborative training approaches that involve both workers and supervisors throughout the process tend to produce materials that are clear, culturally relevant, and practical. This engagement not only improves understanding but also builds trust, encourages buy-in, and contributes to a more lasting safety culture (24, 25). Research consistently indicates that training developed without input from frontline workers or supervisors often does not reflect job-site realities, cultural contexts, or the language needs of the workforce (23, 24). Conversely, when both workers and supervisors participate throughout the process, training is more likely to be clear, culturally appropriate, and feasible to implement (24). Such engagement not only enhances understanding but also builds trust, fosters buy-in, and helps establish a stronger, more sustainable safety culture (25).

The persistently high rates of injuries and fatalities in landscaping and tree care underscore the urgent need for stronger measures to protect workers. At the national level, the Occupational Safety and Health Act (OSHA) of 1970, requires all U.S. employers to provide a workplace free from recognized hazards (26). More specifically, the General Duty Clause (Section 5(a)(1)) and 29 CFR 1910 (General Industry) and 29 CFR 1926 (Construction Standards) establish requirements related to fall protection (Subpart M), electrical safety (Subpart K), machine guarding (Subpart O), and the use of personal protective equipment (Subpart E, 29 CFR) as outlined by the (26). While these federal regulations provide a critical foundation for workplace safety, they are broad in scope and do not fully address the specific hazards and operational realities of landscaping and tree care work. As a result, critical risks such as tree felling, chainsaw operation, exposure to extreme environmental conditions may not be adequately covered or contextualized in standard compliance materials. Such limitations are especially challenging for small businesses, which often lack the administrative capacity or technical resources to adapt general regulations into job-specific training. These challenges disproportionately impact culturally and linguistically diverse workers, many of whom face language barriers, low literacy, and limited prior industry experience, which makes it difficult to access safety training that is structured, accessible, and directly relevant to the job.

To address this gap, this project aimed to develop culturally relevant TBTs that included OSHA-aligned content. The overarching purpose was to create and evaluate TBTs tailored to the realities of landscaping and tree care work, whereby improving hazard awareness, usability, and applicability in small business settings. The study had three specific objectives, (1) design TBTs that reflect real-world hazards and job tasks commonly encountered in landscaping and tree care, (2) assess usability of prototype TBTs evaluating clarity, cultural relevance, and delivery format through iterative feedback from workers and supervisors, and (3), validate effectiveness and utility of the safety messages and recommended practices by examining their perceived value among those directly performing or overseeing the work. Embedding regulatory principles into TBTs for landscaping and tree care occupations creates a scalable framework for evidence-based safety interventions in under-resourced industries.

## Methods

**Figure 1:**
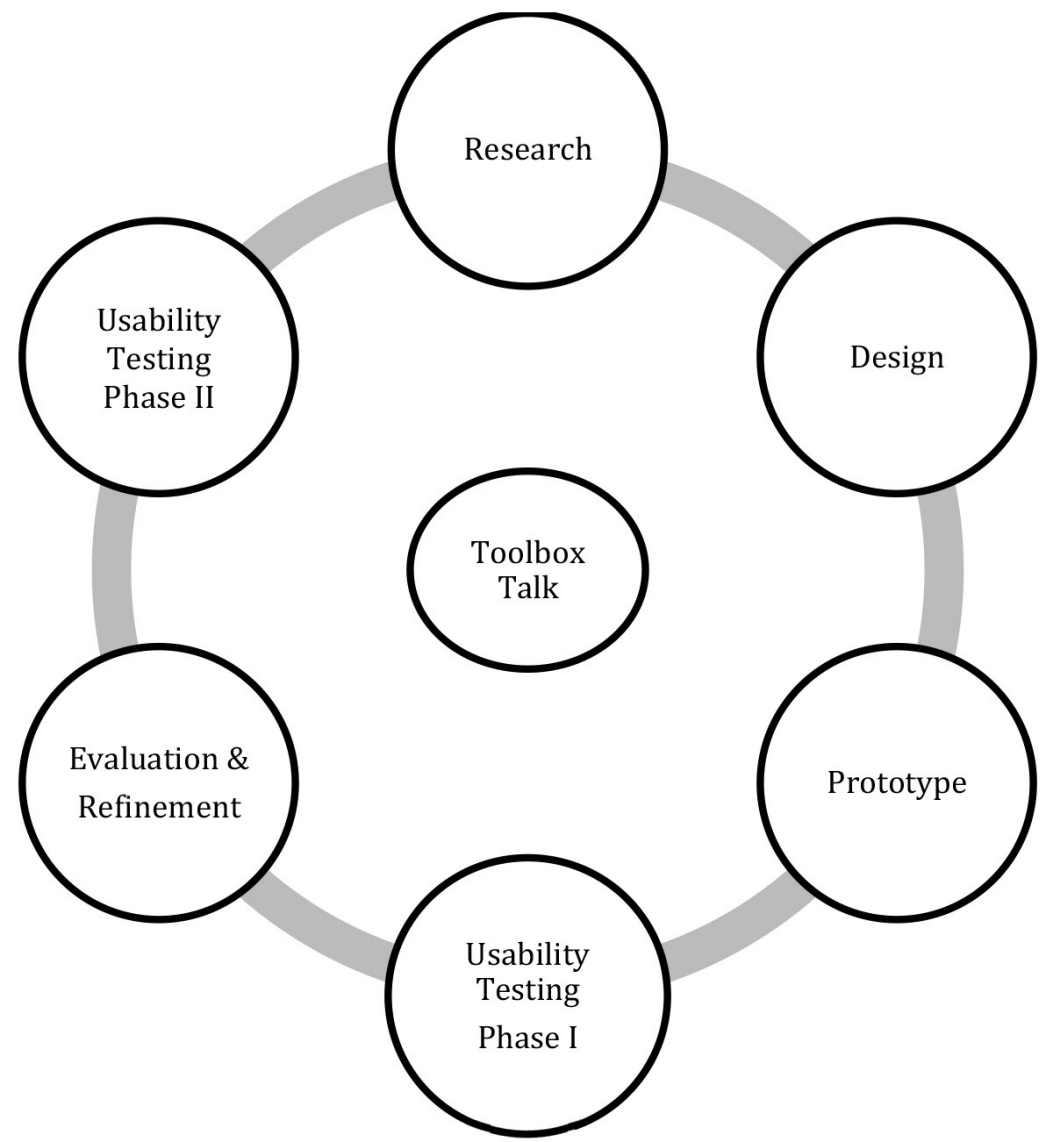
User-centered framework and iterative design approach for developing toolbox talks.

This project used a User-Centered Design (UCD) framework to guide the development and design of Training-Based Tools (TBTs). UCD follows an iterative, multi-phase process aimed at identifying the needs, preferences, pain points, and behaviors of end users throughout different stages of design and implementation (27). The main goal of UCD is to design and improve interactions so that products or services are intuitive, efficient, enjoyable, and meaningful. Within the UCD approach, is usability testing, a method designed to gather user feedback, refine content, and ensure that products or content is relevant, understandable, and practical (28). To make certain that all of the prototype TBTs met the objectives of this study, usability testing was employed with workers and supervisors using an iterative, cyclical process based on continuous engagement (Figure 1).

Participants were recruited through multiple channels, including contacts from prior occupational safety studies, referrals from industry partners, professional networks, and labor organizations. Eligible companies were identified and contacted via formal recruitment letters, emails, and follow-up calls aimed at engaging business owners or site supervisors. To qualify, participants needed to be landscaping and/or tree care workers or supervisors, be at least 18 years old, and able to read and speak either English or Spanish. They were required to participate in a single usability testing session lasting 60 to 90 min.

A purposive sampling strategy was used, following the principle of thematic saturation. This standard in qualitative and usability research indicates that data collection is sufficient once no new themes or insights emerge (29). A sample size of 60 participants was set based on criteria from published literature on usability testing in applied settings (28, 29).

To ensure TBT prototypes effectively addressed more commonly associated workplace hazards and risks, its development was guided by stakeholder input alongside a review of relevant literature and materials. Our search included a wide-range of resources including peer-reviewed studies, government reports, national injury and fatality databases, training resources from various industries and safety organizations. To strengthen the supporting evidence of common fatalities and injuries, the research team reviewed and analyzed more than three decades of National Institute for Occupational Safety and Health (NIOSH), Fatality Assessment and Control Evaluation (FACE) reports (*n* = 93) related to landscaping and tree care incidents (11). From our search, a comprehensive list of 20 topics was identified and prioritized based on input from stakeholder feedback (Supplementary Table).

The initial design of TBT prototype templates was shaped by OSH best practices identified as part of our earlier literature search, and industry examples found on the worldwide web (7, 30). Content language and wording were maintained at a seventh grade reading level and formatted into a Microsoft Word (v10) document.

**Figure 2:**
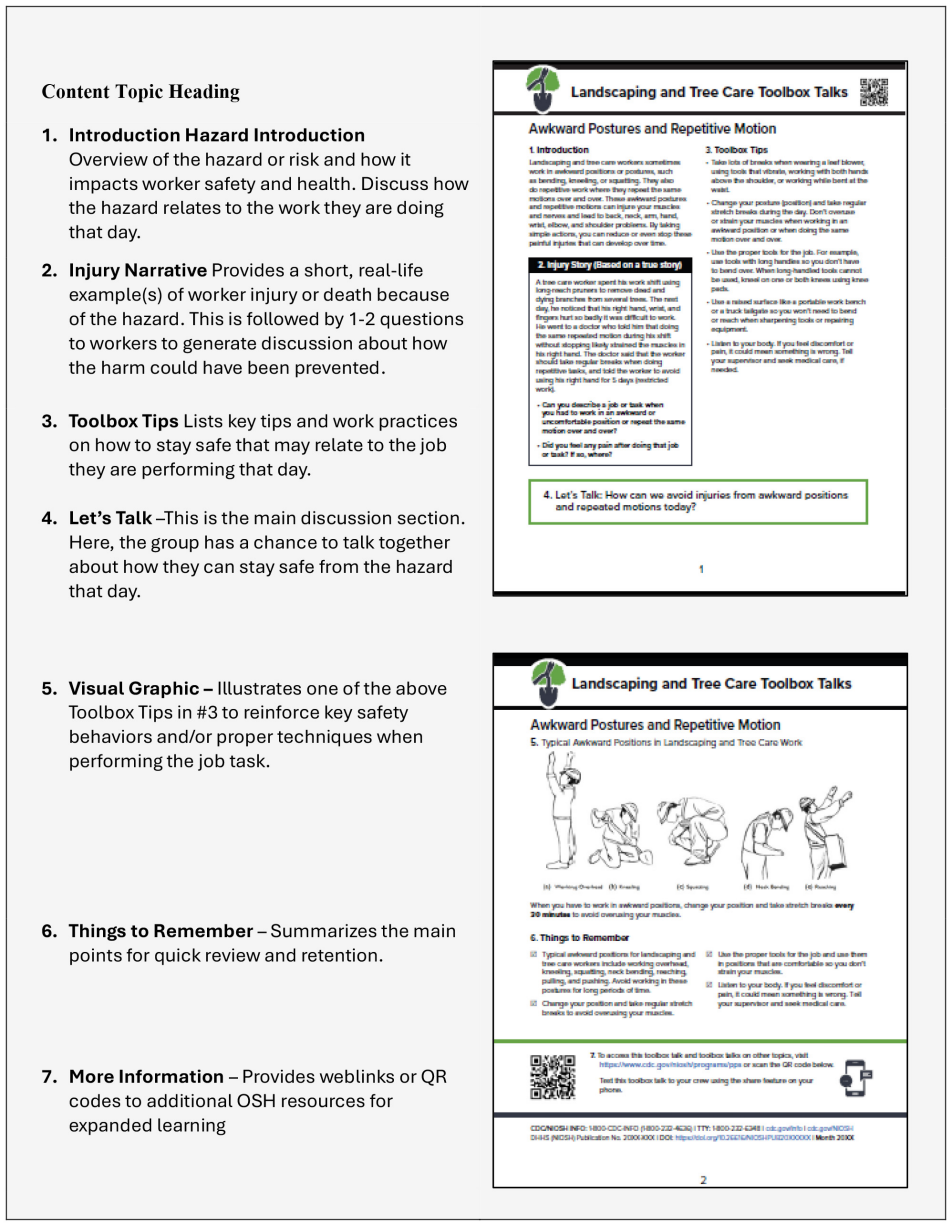
Toolbox talk: design layout and content elements, landscaping and tree care workers.

The format structure of all TBTs developed was consistent throughout the process and were divided into seven, numeric content sections. As shown in Figure 2, the first (1), “Introduction,” section described the relevant occupational hazard or risk, highlighting its relevance and importance to worker safety and health. This section aimed to help supervisors contextualize hazards for workers in relation to their tasks, promoting immediate applicability. In the next section (2), an “Injury Story” was presented, illustrating a real or representative incident involving a worker suffered injury, illness, or fatality attributable to the hazard. Accompanying this narrative were one or two discussion prompts to facilitate workers' reflection on preventive measures for such events. This section invited workers to share similar experiences, fostering open communication, enhancing relatability, and Deepening engagement. In section three (3) “Toolbox Tips,” were presented as key, safety recommendations accompanied by best practices aimed at reducing worker risk. Safety tips were often reflective of regulatory safety standards requirements for similar tasks found in other industries. The “Let's Talk” component (4) offered workers and supervisors an opportunity to communicate with each other and discuss essential safety information, promote safe work behaviors, and increase awareness. This interactive element has been shown to reinforce shared commitment to safety by encouraging workers to talk about safety and hear personal experiences from other workers (31). A simple graphic in section five (5) was included to highlight critical safety points relevant to the specific topic. The “Things to Remember” section (6) aimed to reinforce key safety takeaways, while the final section, (7) “More Information,” featured a scannable QR code and web links directing users to a digital repository of TBTs and additional OSH resources.

Data collection for this research was carried out from March 23, 2023, to January 26, 2024, using a structured usability testing approach. Sessions were led by a member of the research team in group settings involving supervisors and workers from the landscaping and tree care industries, mainly at job sites in North Carolina. Additionally, two sessions were held via secure video conferencing platforms to include participants from other states, with all sessions lasting about 1 hour.

At the start of each session, participants received an overview of the study's goals and an explanation of the usability testing process. Handouts and materials were provided in both English and Spanish, based on participant preference, with Spanish-language sessions facilitated by a native Spanish-speaking moderator. Data collection involved recording responses manually during in-person sessions and transcribing them from audio recordings in remote sessions. Participants reviewed multiple TBT prototypes during each session, and the collected feedback was used to guide iterative improvements aimed at increasing the clarity, usability, and overall effectiveness of the TBTs.

The data collection process occurred in two distinct phases, (i) initial testing and (ii) validation. Each phase of testing with participants was guided by a three-part questionnaire, based on well-established usability testing principles (27). Prior to testing with the study sample, the three-part questionnaire was pre-tested with a small group of non-landscapers and landscapers for clarity and content.

Part I of the questionnaire was general background information that included open-ended and multiple-choice questions about participants' job roles and their experiences with occupational safety and health. Topics covered included job classification, tools used, employer type, work hours, length of employment in landscaping or tree care, history of work-related injuries, and whether they had participated in any OSH training within the past year.

Part II was the TBT Interaction and “Think-Aloud” Evaluation section. In this part of the questionnaire, participants interacted with a printed copy of the TBT prototype. To replicate an actual job-safety training scenario, a supervisor was given a TBT and asked to read aloud to workers in a group setting while participants followed along with their personal copy. At the conclusion of the TBT reading by the supervisor, a series of “think-aloud” and follow-up questions were presented to participants by the researcher. These questions were designed to assess comprehension, usability of the training materials, and relevance to workers daily tasks. This approach also was intended to evaluate communication clarity and the practical use of the training materials and content.

Part III was the Post-Testing Feedback section. In this final part, open-ended questions were asked to gather opinions and perceptions of the training experience. Areas of feedback included overall effectiveness, clarity, and flow of the TBTs, comparisons with any previous or current training methods, relevance to daily work, and suggestions for improvements.

This study received ethical approval from the East Carolina University Institutional Review Board (UMCIRB #21-002270) before the initiation of data collection.

Usability testing sessions took place from March 23 to May 31, 2023, involving 22 participants, which accounted for 36.1% of the total sample, across six sessions at three different job sites. The primary objective of Phase I was to gain user insights into the initial layout and design of TBTs, as well as to identify usability barriers—referred to as “pain points” in the structure and delivery of these TBTs. In usability research, “pain points” denote recurring obstacles, inefficiencies, or frustrations that impede a user's engagement or understanding (32). Throughout this phase, all participants were provided with a prototype TBT, while a supervisor simulated a safety TBT in the field with workers. By employing direct observation, structured moderator prompts, and iterative feedback, researchers documented specific usability challenges, including unclear terminology, inconsistencies in visual layout, and navigation difficulties. Revisions to the TBT design and content were made between sessions, enabling subsequent participants to test the improved versions.

Descriptive statistics were computed using SPSS (IBM, version 28.1) to summarize participant demographics and occupational characteristics. Qualitative data, including field notes, open-ended responses from usability testing, and transcripts of virtual interviews were reviewed iteratively to identify patterns and extract valuable insights related to content comprehension and usability.

All qualitative materials were uploaded into NVivo (version 12) for thematic analysis. Recurring phrases, terms, and expressions were systematically coded to uncover common usability themes and identified areas for improvement. Word frequency queries and visualizations, such as word clouds, were generated to aid in the identification of prominent concepts. This integrated approach provided both quantitative and qualitative perspectives, facilitating a comprehensive understanding of how workers engage with and interpret the TBTs, while also informing subsequent refinements to enhance clarity, relevance, and applicability.

## Results

As shown in Table 1, out of a total sample of 60 participants (*N* = 60), 18 individuals (30.6%) took part in virtual testing, while 42 participants (69.4%) attended in person at the job site. Most respondents (61.1%) identified as workers, with the remaining 38.9% holding supervisory roles. Regarding job responsibilities, nearly half (47.2%) performed tasks in both landscaping and tree care, while an additional 30.6% focused only on landscaping, and 22.2% specialized in tree care. The reported job tasks varied, with mowing and trimming noted by 66.7% of participants, cutting and pruning by 27.8%, and operating heavy equipment by 5.6%. Participants worked for a variety of organizations, including nationally operated companies (30.6%), independently owned businesses (25.0%), local government agencies (19.4%), universities (8.3%), and regional hospitals (8.3%). A significant portion (69.4%) were employed by organizations with 25 or more employees. Additionally, 25.0% worked for small businesses with 14 or fewer staff members, and 5.6% belonged to companies with between 15 and 24 employees. Most participants (55.6%) reported working 21–40 h per week, while 41.7% worked over 40 h. Field experience varied: 83.3% were employed for 1 to 2 years, 11.1% for over 2 years, and 5.6% for less than a year. Approximately one-third reported having experienced a work-related injury. Just over half (52.8%) had received occupational safety and health (OSH) training within the past year, while 47.2% had not received training.

**Table 1:** Number of participants and work characteristics, landscaping, and tree care workers.

**Characteristic**	***n***	**(%)**
Total participants tested (*N*)	60	100.0
Virtual participants	18	30.6
In-person participants	42	69.4
**Primary job classification**		
Worker	37	61.1
Supervisor	23	38.9
**Primary job category**		
Landscaping	18	30.6
Tree care	13	22.2
Both landscaping and tree care	28	47.2
^*^ **Primary job tasks**		
Mowing and trimming (i.e., mowers, weed trimmers, edger's, hedge trimmers)	40	66.7
Cutting and pruning (i.e., chainsaws, climbing gear)	17	27.8
Heavy equipment (i.e., backhoes, bucket trucks, earth movers)	2	5.6
**Employer**		
Local government	12	19.4
Community living complex	3	5.6
Primary and secondary school	2	2.8
University	5	8.3
Regional hospitals	5	8.3
National groundskeeping and/or tree care businesses	18	30.6
Independently owned/operated	15	25.0
**Employer size of landscaping/tree care workforce**		
25 or more workers	42	69.4
15–24 workers	3	5.6
14 or less workers	15	25.0
**Hours worked per week**		
Less than 20	2	2.8
Between 21 and 40	33	55.6
More than 40	25	41.7
**Length of time working in landscaping and/or tree care**		
Less than 1 year	3	5.6
Between 1 and 2 years	50	83.3
More than 2 years	7	11.1
**Ever been injured performing landscaping or tree care work**		
Yes	20	33.3
**OSH training within the past 12 months (at current job)**		
Yes	32	52.8
No	28	47.2

^*^All participants reported performing multiple job tasks.

Initial testing identified significant structural and delivery challenges, particularly regarding supervisors' uncertainty about how to effectively facilitate Toolbox Talks (TBTs) (see Table 2), supervisors faced challenges in following the outline format, leading to inconsistent delivery. Sometimes, external cues were needed to maintain flow (Pain Point #1). To fix this, the research team reorganized the TBT format by changing the font size of headings and numbering the sections. The updated materials were tested with supervisors to improve clarity and usability. A Supervisor User Guide was also created to provide simple instructions, delivery tips, and suggestions for customizing the talks to different work crews. This guide was tested during mid-phase sessions and refined based on user feedback.

**Table 2:** Summary of usability barriers and design improvements for toolbox talks in landscaping and tree care, Phase I (*n* = 22).

**User “Pain Points”**	**Description**	**Corrected actions**
Pain Point #1: Difficulty navigating TBTs during delivery	Supervisors had trouble presenting the TBTs smoothly, often needing prompts and losing their place in the document.	Reformatted the TBT layout and developed a concise *Supervisor User Guide*. Conducted follow-up usability testing with supervisory personnel.
Pain Point #2: Excessive test or “wordiness”	Content was generally understandable, but several participants noted that the documents were too lengthy or repetitive.	Prioritized key points, reduced redundancy, and eliminated non-essential wording to improve readability.
Pain Point #3: Visuals lacked clarity or effectiveness	Participants requested clearer, more impactful illustrations, including side-by-side depictions of correct and incorrect task performance.	Updated illustrations for clarity and added comparative visuals to reinforce correct practices where relevant.
Pain Point #4: Preference for digital access	Younger participants and supervisors expressed a preference for digital formats and on-demand safety information.	Added a “More Information” section with QR code and OSH resources and relevant OSH websites.
Pain Point #5: Language and terminology issues	Some Spanish-speaking workers found some words or terms (e.g., “landscaper”) as being unfamiliar, or confusing due to false cognates. Ancillary, Spanish workers had difficulty finding relevant OSH resources noted.	Revised terminology based on user input for cultural relevance. Highlighted accessible, bilingual OSH resources via embedded links.

Concerns about excessive “wordiness” (Pain Point #2) were identified, prompting the team to streamline content by emphasizing key messages and removing redundancies. Workers preferred more visual elements (Pain Point #3), especially illustrations showing safe vs. unsafe practices. These were addressed by updating graphics and adding instructional visuals. Some participants wanted the ability to access TBT content on their own (Pain Point #4), showing interest in digital formats and QR codes. Follow-up sessions assessed how well this improved accessibility and engagement.

Lastly, language clarity was a concern for Spanish-speaking participants unfamiliar with terms like “landscaper” (Pain Point #5). To improve understanding, “gardener” was used instead of “landscaper” in the Spanish versions of the TBTs, and plans were made to include additional links to additional OSH information.

The iterative process of usability evaluation in Phase II provided important insights into the revised Toolbox Talks (TBT) prototypes, especially regarding content clarity, relevance, and usability. Validation sessions, summarized in Table 3, showed that the updated TBTs were generally seen as accessible and effective at delivering key Occupational Safety and Health (OSH) information. Participants demonstrated a thorough understanding of the TBT content, effectively navigating the materials' structure, identifying relevant hazards, and confidently recalling recommended protective measures.

**Table 3:** Summary of content validation and recurring themes of toolbox talk prototypes, Phase II (*n* = 38).

**Content**	**Description of recurring themes**
Clarity and comprehension	Participants demonstrated a clear understanding of the TBT content section areas. Workers and supervisors validated key safety hazards concerns and recommended protective actions. The “True Story” injury narratives were widely regarded as meaningful, engaging, realistic, and personally relatable.
Task relevance and safety actions	Many participants were highly engaged during validation sessions, frequently sharing personal stories from their job experiences that demonstrated the relevance of the TBT content. These narratives often reinforced the applicability of the recommended safety actions and affirmed the value of TBTs in promoting safe work practices.
Delivery preferences and practical use	Supervisors expressed confidence in delivering TBTs using the revised format. Participants appreciated the clear layout and practical tips. Accessibility options continued to vary, with older workers preferring print copies of TBTs, while most younger workers requested having digital access (e.g., QR codes).
User engagement and familiarity	Workers employed by larger structured organizations and institutions found TBTs to be well aligned with their existing safety training practices and readily accepted them. In contrast, those from smaller, independent operations, were appreciative of the content, but were generally less familiar with the TBT format and expressed a need for clearer introductory explanations.
Cultural relevance and language	Most Spanish-speaking and foreign-born participants found the terminology and graphics primarily culturally appropriate. However, minor inconsistencies in some Spanish-language phrasing and English phrasing remained and noted for correction.
Graphics and visual aids	Visuals were generally helpful for reinforcing key safety concepts. However, some terminology in illustrations did not fully match field language or TBT text, prompting revisions for alignment and clarity.

Feedback indicated a consensus among both workers and supervisors about the accuracy and relevance of the information, which matched the safety challenges they face in their jobs. Notably, the “True Story” segments resonated with participants, who found these narratives based on real experiences to be especially impactful. These segments helped put safety messages into context, reinforcing their importance.

During validation sessions, participants often shared personal experiences related to the risks and safety practices shown in the TBTs. These spontaneous stories highlighted the practical relevance of the materials and supported the suggested safety behaviors. The active participation of workers suggested a strong connection with the TBT content, highlighting its relevance to everyday work. From a practical perspective, supervisors felt more confident in delivering the revised TBTs, noting that the structured format made discussions more effective. Participants valued the inclusion of practical tips in each section. However, preferences for how the TBTs were delivered varied greatly among demographic groups; older participants preferred printed materials, while younger individuals favored digital access via QR codes for on-demand viewing on mobile devices.

The success of the TBT format also depended on organizational context. Employees in larger, more structured companies found the TBTs fit well within their existing safety training programs, whereas those in smaller operations, while appreciating the content, suggested that future versions should have a clearer introductory section to help users get familiar with the format.

Language and cultural relevance were key themes in the feedback. Spanish-speaking and foreign-born workers generally found the terminology and visuals culturally appropriate. However, some inconsistencies in phrasing between the English and Spanish versions were noted, along with terminology differences common in the field. These issues were carefully documented and addressed to improve cultural and linguistic clarity in future updates.

Finally, while participants appreciated the use of graphics to clarify safety concepts, they pointed out several cases where the terminology used in visuals didn't match the language in the accompanying text. Addressing these discrepancies was a priority for researchers, aiming to enhance overall clarity and consistency across the TBT materials.

## Discussion

This project accomplished three core objectives. First, we created prototype TBTs that addressed real hazards and daily job tasks faced by landscaping and tree care crews. We ensured relevance by conducting on-site observations, following established industry best practices, and most importantly, involving direct input from workers and supervisors. As a result, the materials were rooted in lived experience, boosting their practical usefulness in the field. Second, we tested the effectiveness of the TBTs through multiple feedback sessions with workers and supervisors from small landscaping and tree care companies. These sessions identified challenges related to layout, terminology, and visual presentation, as well as elements that participants found helpful. In response, we made key modifications: bilingual formats, clearer language, and inclusion of relatable incident stories. These changes made the materials easier for both workers and supervisors to use, which directly increased participation during safety talks. Finally, we evaluated how well the revised TBTs supported hazard awareness and safe work practices. Workers reported that the TBTs were easier to navigate and more useful than the generic safety handouts they had used previously (if any). Supervisors felt more confident presenting the TBTs and leading discussions, noting increased worker participation. These findings support previous research by (4, 25, 33), which show that involving workers in developing training tools improves communication and enhances safety message impact. This is especially important because most injuries and fatalities occur in small businesses with limited training resources (4, 34). The study also assessed how effective the TBTs were in helping workers understand and apply safety information. Workers said the materials were easier to follow and more useful than standard safety handouts. Supervisors reported feeling more confident leading safety talks and saw greater participation from their crews. Overall, the findings suggest that involving workers in the development process fosters communication that can strengthen daily safety practices, especially in small business settings.

Limitations of the study include that feedback was self-reported and often collected in group settings with supervisors present, which may have influenced some responses. For example, some participants might have held back comments due to reasons like criticism or fear of retaliation (35). Nonetheless, moderators emphasized confidentiality and encouraged honest discussion (36). Additionally, the project did not track long-term use of the TBTs or their impact on injury rates, leaving room for future research. The broader implications demonstrate that user-centered design and participatory evaluation can produce practical safety tools for small, high-risk industries. In this case, OSHA-aligned requirements, covering hazard communication, fall protection, PPE, and training were incorporated into short, culturally adapted safety talks that crews could realistically use on the job. Future work should examine how these TBTs perform over time, influence actual safety behaviors, and determine the best ways to deliver them in print and digital formats. Partnering with trade associations, insurers, and state consultation programs could help expand their adoption, especially among small employers with limited resources. Extending the approach to other language groups and tailoring content for different cultural contexts could further increase reach and effectiveness.

In conclusion, integrating OSHA-informed safety requirements into short, culturally relevant Toolbox Talks offers a practical way to bridge the gap between policy and daily practice in landscaping and tree care. Developed through an iterative, worker-centered process, these talks address hazard identification, fall protection, PPE, and training, while being adaptable to the language, culture, and workflow of diverse crews. Although these occupations are mainly unregulated, this study shows that training can still be policy-guided and effective in meeting key OSHA standards. For small businesses, adopting such TBTs provides a scalable way to improve hazard communication, foster a stronger safety culture, and reduce injury and death risks. With ongoing use and further evaluation, this approach could serve as a model for enhancing safety in other high-risk, under-resourced industries, occupational sectors.

## Data Availability

This study was conducted under a NIOSH-funded cooperative agreement; however, the study team retains custodial responsibility for all collected data. Although NIOSH has the right to review and access project data for oversight purposes, it does not maintain, store, or distribute this dataset. Accordingly, the study data are not available from NIOSH. De-identified data may be made available from the corresponding author upon reasonable request and with appropriate institutional approvals.
