# Supplementary material for: Bridging the occupational policy to practice gap: user-centered designed toolbox talks for landscaping tree care
Source: Front Public Health. 2025 Nov 28;13:1690149. doi: 10.3389/fpubh.2025.1690149 (PMC12699983; doi:10.3389/fpubh.2025.1690149)
Supplement: Supplementary file 1 [file Table_1.docx]

| **Toolbox Talk List of Topics: Landscaping and Tree Care Workers** | |  |
| --- | --- | --- |
| No.# | Toolbox Talk Topic/Title |  |
| 1 | Awkward Positions and Repeated Motions* |  |
| 2 | Chainsaws |  |
| 3 | Cold Weather* |  |
| 4 | Communication for Safety |  |
| 5 | Falls from Heights |  |
| 6 | Hand and Power Tools |  |
| 7 | Heavy Lifting * |  |
| 8 | Hot Weather* |  |
| 9 | Ladder Safety |  |
| 10 | Lawn Mowing Projectiles |  |
| 11 | Lightning* |  |
| 12 | Motor Vehicles: Road Safety |  |
| 13 | Noise and Hearing Protection |  |
| 14 | Pesticides |  |
| 15 | Riding Mowers: Roll-Overs |  |
| 16 | Slips, Trips, and Falls |  |
| 17 | Stinging Insects, Spiders and Ants |  |
| 18 | Struck-By Hazards and Falling Objects* |  |
| 19 | Trenching and Irrigation |  |
| 20 | Wood and Brush Chippers |  |
| *Includes Spanish versions | |  |
